# Blind Brush Biopsy: Quantification of Epstein–Barr Virus and Its Host DNA Methylation in the Detection of Nasopharyngeal Carcinoma

**DOI:** 10.34133/research.0475

**Published:** 2024-09-24

**Authors:** Caoli Tang, Xizhao Li, Yumeng Zhang, Ting Zhou, Xiaojing Yang, Ying Liao, Tongmin Wang, Yongqiao He, Wenqiong Xue, Weihua Jia, Xiaohui Zheng

**Affiliations:** ^1^ State Key Laboratory of Oncology in South China, Guangdong Key Laboratory of Nasopharyngeal Carcinoma Diagnosis and Therapy, Guangdong Provincial Clinical Research Center for Cancer, Sun Yat-sen University Cancer Center, Guangzhou 510060, P. R. China.; ^2^ School of Public Health, Sun Yat-sen University, Guangzhou 510080, P. R. China.

## Abstract

**Background:** The nasopharyngeal brush sampling can effectively collect samples from the nasopharynx. The blind brush sampling does not require the guidance of endoscopy, which is favorable for implementation and dissemination in the community. This study explored methylation markers for nasopharyngeal carcinoma (NPC) at both Epstein–Barr virus (EBV) and its host genome levels, aiming to construct a blind brushing diagnostic method. **Methods:** EBV DNA capture and methylation sequencing and GEO Illumina 450K methylation array data were used respectively for the discovery of EBV and host methylation markers. The diagnostic method was built in training cohort (*n* = 347) and validated in an independent validation cohort (*n* = 155). **Results:** A total of 1 EBV methylation marker (BILF2) and 6 host methylation markers (ITGA4, IMPA2, ITPKB, PI9, AMIGO2, and VAV3) were identified. Both EBV and host methylation markers were almost exclusively detected in NPC samples, with negligible detection in control samples. In validation cohort, the diagnostic method that included only the EBV BILF2 marker showed a sensitivity and specificity of 80.22% and 98.44%, respectively. When combining the EBV-derived marker BILF2 with the host-derived marker IMPA2, the diagnostic method’s sensitivity increased to 84.62%, while the specificity remained unchanged (IDI = 4.4%, *P* = 0.0419). **Conclusion:** Overall, the blind nasopharyngeal brushing diagnostic method, combining EBV and host methylation markers, showed great potential in NPC detection and could promote its application in nonclinical screening of NPC.

## Introduction

Nasopharyngeal carcinoma (NPC) is a highly invasive and metastatic tumor. Its occurrence shows obvious familial and regional clustering, being prevalent in the south China region [1]. The prognosis for early-stage NPC patients is favorable, with a 5-year survival rate of up to 95% [2]. However, due to the lack of obvious early symptoms, most of the NPC patients have already progressed to the advanced stages by the time of their initial diagnosis [3]. Therefore, early detection of NPC is of great importance. In endemic regions, the development of the vast majority of NPC is strongly associated with Epstein–Barr virus (EBV) infection. So far, various EBV-related biomarkers have been used for NPC early screening, including EBV serologic antibodies [4,5] and plasma DNA [6,7].

Methylation modifications of CpG islands within the transcriptional regulatory regions of genes play important roles in normal physiology and disease development. Studies have demonstrated the presence of aberrant hypermethylation in NPC tissues [8]. The promoter regions of numerous tumor suppressor genes (TSGs), including DAPK1, RASSF1A, and CDKN2A, undergo hypermethylation, resulting in suppressed expression. Additionally, signaling pathways including Wnt, mitogen-activated protein kinase (MAPK), and transforming growth factor-β (TGF-β) are affected, thus influencing the initiation and progression of tumors [9]. Many research teams have investigated the diagnostic utility of genes with aberrant methylation modifications in NPC. Their findings suggest that the aberrant methylation of TSGs can aid in diagnosing NPC [10,11].

Some proteins expressed by EBV, such as LMP1 and LMP2A, partially mediate the hypermethylation modifications of genes in NPC by influencing the activity of DNA methyltransferases (DNMTs) [12]. The epigenetic modifications of EBV genome also play significant physiological roles, including regulating the EBV life cycle, evading immune surveillance, and driving host cell carcinogenesis [13]. In NPC patients’ samples, the EBV genome was similarly aberrantly hypermethylation modification. Our research further revealed that this phenomenon was common in other EBV-related tumors. However, there were some differences among these tumors [14]. In addition, we found that EBV in the saliva of NPC patients exhibits hypermethylation, whereas in healthy control subjects, it shows hypomethylation [15].

We have previously developed a nasopharyngeal brush sampling method and evaluated several types of biomarkers, such as EBV DNA load and EBV miRNA, suggesting their potential utility in NPC detection [16–19]. Compared with nasopharyngeal brush sampling, the blind brush sampling method does not require the guidance of endoscopy, which is favorable for implementation and dissemination in the community. Our previous findings suggest that blind brush sampling is an alternative to nonblind brush sampling [20]. The EBV C promoter (Cp) region has a significant methylation difference in blind brushing samples from NPC and control groups. In most NPC patients, this region is predominantly methylated, while almost all control samples have a nonmethylated modification. Diagnostic model constructed using the methylation level of Cp region has a sensitivity of 95.9% and a specificity of 91.7% in nonblind brushing samples, and the specificity increased to 94.6% in blind brushing samples, but the sensitivity was only 82.8%, still better than EBV DNA load [20]. The possible reason for the decreased sensitivity in the blind brushing samples may be due to incomplete sampling during some blind brushing procedures, as there is no nasopharyngeal endoscopy guidance. Consequently, fewer or even no samples containing EBV nucleic acid fragments are collected, resulting in a lower detection rate. To address this issue, this study proposes enhancing diagnostic sensitivity by integrating methylation markers from host genome sources.

In this study, differential methylation biomarkers between NPC and control blind nasopharyngeal brushing samples were explored. Through EBV DNA capture and methylation sequencing, we rescreened differentially methylated EBV genes in blind brushing samples. Host differentially methylated genes were identified through analysis of Illumina 450K methylation array data. To enhance the performance of NPC diagnostic methods, a blind brush biopsy diagnostic method for NPC was developed by integrating differential methylation biomarkers from both EBV and host origins.

## Results

### Discovery of EBV DNA methylation markers to distinguish NPC from control

A total of 7,125 CpG sites were successfully sequenced in 1 control sample and at least 3 NPC samples. For the same CpG site, the methylation level of the control sample was defined as the methylation level at that site in one successfully sequenced control sample. The methylation level of the case samples was defined as the average methylation level at that site among 6 successfully sequenced NPC samples. Our previous studies have found significant differences in methylation modification at the 11,029-base pair (bp) site located in the EBV Cp region between NPC and control saliva samples [15]. In the majority of NPC patient samples, methylation modification predominates at this site, while almost all control samples show no methylation modification. This phenomenon has also been validated in nasopharyngeal brushing samples [20]. Therefore, this study preferred sites with extremely low methylation levels in control samples (methylation level < 20.00%) and extremely high levels in case samples (methylation level > 70.00%). The regions enriched with differential methylation sites (containing ≥5 adjacent differential methylation sites) were selected as candidate detection regions. Two regions have been selected: one located between 10,243 and 10,608 bp in the intergenic region, and the other within the BILF2 gene spanning from 137,999 to 138,149 bp. Compared to the previously discovered Cp region, the methylation levels of the sites within these 2 regions exhibited greater differences between groups (Fig. S1).

**Fig. 1.**
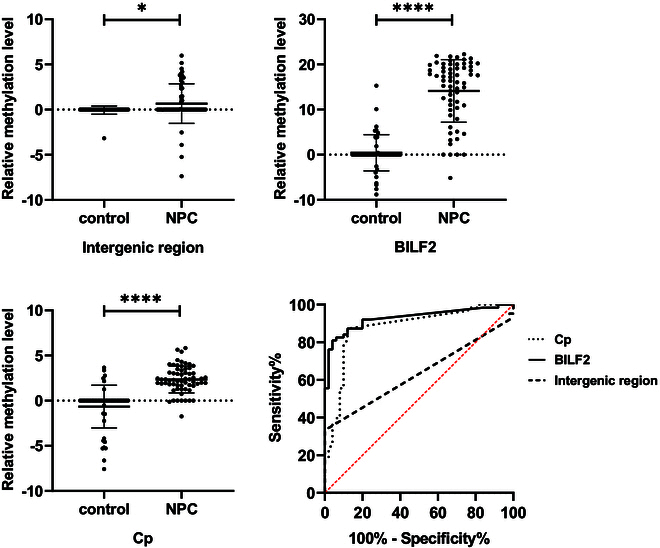
The relative methylation levels and modeling ROC of intergenic region, BILF2, and Cp DMRs.

In order to validate the EBV-targeted bisulfite sequencing results, we collected 63 NPC samples and 50 control samples, and performed quantitative methylation-specific polymerase chain reaction (PCR) (qMSP) testing targeting the above 3 regions. Although the relative methylation levels of all 3 regions were statistically different between case and control groups, the detection rate in the intergenic region (control 2.00%, NPC 41.27%) was the lowest, and the detection rate of BILF2 (control 40.00%, NPC 93.65%) was slightly higher than Cp (control 36.00%, NPC 90.48%). Compared to the diagnostic model constructed with Cp, the BILF2 model had a higher area under the curve (AUC) (0.929), with an improvement in integrated discrimination improvement (IDI) (0.126), while intergenic region had the lowest AUC (0.634), with a decrease in IDI (−0.442) (Table 1 and Fig. 1). In summary, the BILF2 methylation indicator had the best performance.

**Table 1.** qMSP detection of 3 regions in NPC and control brushing samples

	Intergenic region	BILF2	Cp
Detection rate (%)			
Control	1/50 (2.00)	20/50 (40.00)	18/50 (36.00)
NPC	26/63 (41.27)	59/63 (93.65)	57/63 (90.48)
Relative methylation level (‾X ± S)			
Control	−0.06 ± 0.45	0.42 ± 4.02	−0.66 ± 2.37
NPC	0.67 ± 2.20	14.12 ± 6.94	2.35 ± 1.48
*P*	0.023	<0.001	<0.001
Diagnostic model performance (95% CI)			
AUC	0.634 (0.531, 0.736)	0.929 (0.880, 0.978)	0.879 (0.811, 0.948)
IDI	−0.442 (−0.536, −0.347)	0.126 (0.036, 0.216)	Ref

CI, confidence interval

### Discovery of host DNA methylation markers to distinguish NPC from control

Host source candidate markers were selected based on the analysis of Gene Expression Omnibus (GEO) datasets. Two screening strategies were used. The one was a candidate strategy, which analyzed CpG sites within the regions of candidate genes reported in previous studies to screen for differentially methylated regions (DMRs). The other was reanalysis strategy, which reanalyzed all data to screen for differentially methylated genes.

#### Candidate strategy

Based on literature and patent review, we summarized 16 candidate genes: RASSF1, DAPK1, CDKN2A, CDH1, RARB, CDKN2B, ITGA4, ZNF671, WIF1, ITGA9, SHISA3, SEPTIN9, DACT1, NFATC2, SFRP5, and ZMYND10.

The criteria for analyzing hypermethylated sites in NPC were as follows: (a) the difference in methylation levels between the NPC and control group in GSE52068 was ≥0.2, with an adjusted *P* < 0.05; (b) the difference in methylation levels between the groups in GSE62336 was ≥0, with an adjusted *P* < 0.05. DMRs were obtained as follows. Based on the annotation information from the Illumina 450K methylation array, the chromosome and base position of each successfully detected site were extracted and sorted in sequence. If there were 5 or more hypermethylated sites on the same chromosome and adjacent in sorted position, they were merged into a DMR.

**Fig. 2.**
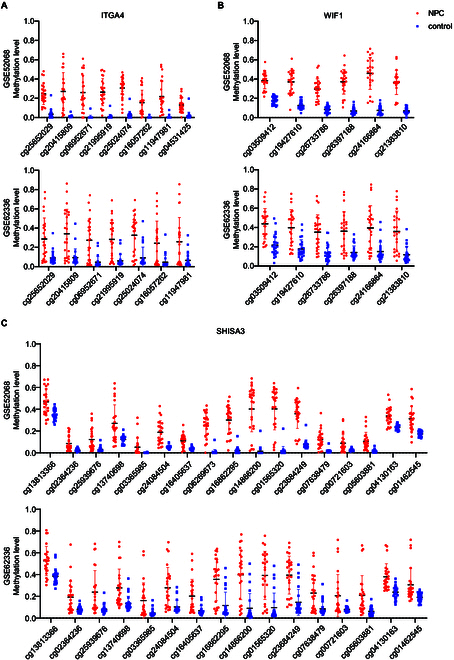
Intergroup comparison of methylation levels of CpG sites within DMRs on ITGA4 (A), WIF1 (B), and SHISA3 (C) genes in GSE52068 and GSE62336.

According to the above criteria, 3 differentially methylated genes were screened including ITGA4, WIF1, and SHISA3. The DMRs for these 3 genes were as follows: NC_000002.11: 182,321,912 to 182,322,163 bp, NC_000012.11: 65,514,821 to 65,515,670 bp, and NC_000004.11: 42,399,499 to 42,400,158 bp. Comparison of methylation levels of sites within each DMR region between NPC and control is shown in Fig. 2.

#### Reanalysis strategy

The data from GSE52068 and GSE62336 were individually reanalyzed to identify hypermethylated sites, utilizing the following criteria. (a) Statistical test for difference in methylation levels of CpG sites between NPC group and noncancer control group in GSE52068 with a statistic of ≥10. In GSE62336, the statistical test for intergroup methylation differences had an adjusted *P* value of <0.05. (b) The average methylation level of CpG sites in the control group of GSE52068 was <0.01, or the average methylation level of CpG sites in the control group of GSE52068 was ≥0.01 but <0.03, and <0.1 in the control group of GSE62336.

DMRs were obtained as follows. Based on the annotation information from the Illumina 450K methylation array, the chromosome and base position of each successfully detected site were extracted and sorted in sequence. If there were 2 or more hypermethylated sites on the same chromosome and adjacent in sorted position, they were merged into a DMR.

**Fig. 3.**
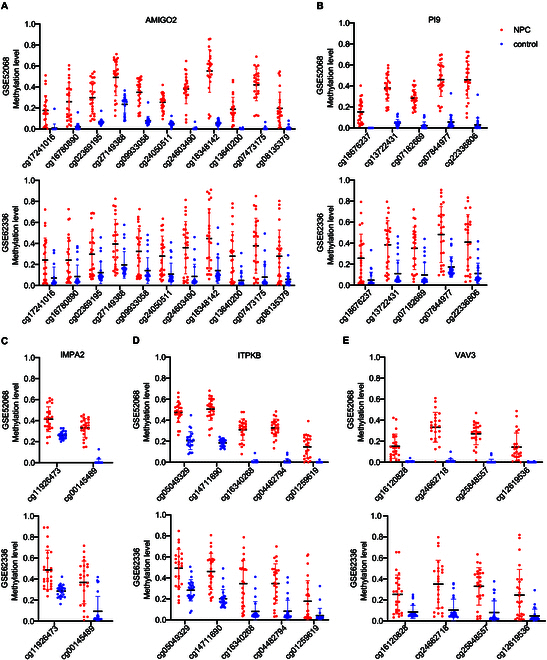
Intergroup comparison of methylation levels of CpG sites within DMRs on AMIGO2 (A), PI9 (B), IMPA2 (C), ITPKB (D), and VAV3 (E) genes in GSE52068 and GSE62336.

According to the above criteria, 5 differentially methylated genes were screened including AMIGO2, PI9, IMPA2, ITPKB, and VAV3. The DMRs for these 5 genes were as follows: NC_000012.11: 47,473,928 to 47,474,281 bp, NC_000006.11: 2,903,436 to 2,903,756 bp, NC_000018.9: 11,980,559 to 11,980,832 bp, NC_000001.10: 226,925,108 to 226,925,381 bp, and NC_000001.10: 108,507,448 to 108,507,823 bp, respectively. Comparison of methylation levels of sites within each DMR region between NPC and control is shown in Fig. 3.

To preliminarily validate the analysis results, we performed PCR amplification of the above DMRs in 8 NPC and 8 control samples and assessed the methylation status of CpG sites within the product through Sanger sequencing. According to the Sanger sequencing results, the DMRs on genes ITGA4, IMPA2, and ITPKB were methylated in NPC samples, while they were unmethylated in the control samples (Fig. S2). The DMRs on gene SHISA3 were unmethylated in both NPC and control samples (Fig. S3). The DMRs on genes PI9, AMIGO2, and VAV3 were methylated in NPC samples, while sequencing failed in control samples (Fig. S4). In addition, the DMR on WIF1 gene was excluded due to amplification failure. Therefore, ITGA4, IMPA2, ITPKB, PI9, AMIGO2, and VAV3 genes were selected as candidate methylation markers.

### Verification of methylation markers and construction of diagnostic methods in training cohort

**Fig. 4.**
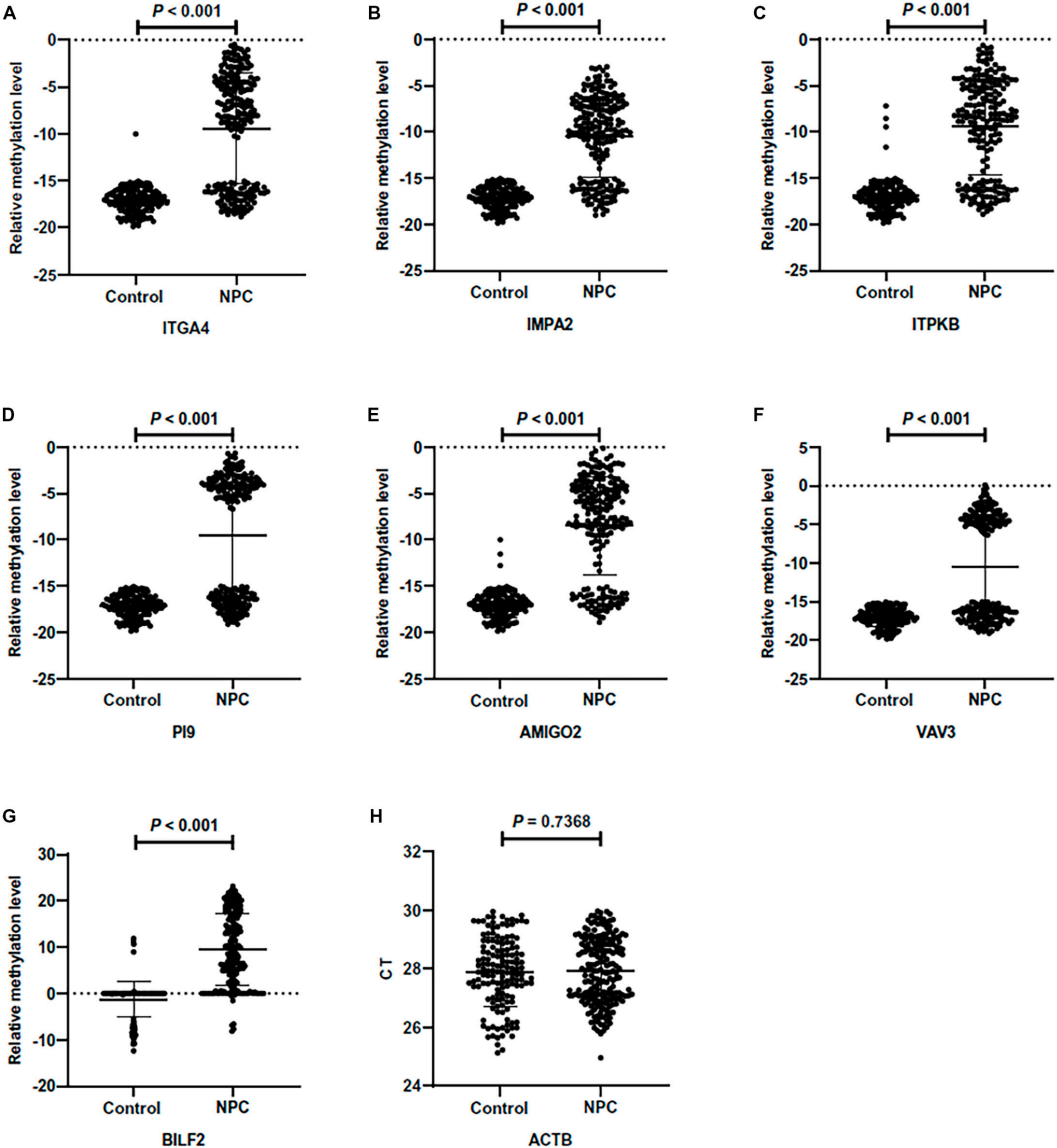
Comparison of relative methylation levels of candidate methylation markers [ITGA4 (A), IMPA2 (B), ITPKB (C), PI9 (D), AMIGO2 (E), VAV3 (F), and BILF2 (G)] between case-controls in the training set and comparison of CT values of internal reference gene ACTB (H).

A total of 452 subjects were recruited including 173 controls and 279 NPC patients. After excluding disqualified samples, the remaining 347 brushing samples (145 controls and 202 NPC) were included in the analysis. There was no significant difference of CT_ACTB_, indicating no difference in sample quality between the groups (Fig. 4). For the EBV marker BILF2, methylated product (relative methylation level > 0) predominated in NPC samples, while unmethylated product (relative methylation level < 0) was predominant in control samples (Table 2). As for host source methylation markers, the methylated products of host markers were almost undetectable in control samples, whereas they were detectable in the majority of NPC samples (Table 2). There was no difference in the detection between samples of different genders (Table S1 and Fig. S5). Therefore, the relative methylation levels of each candidate methylation markers were statistically different between NPC and control samples.

**Table 2.** Candidate marker methylation detection in the training set (*n* = 347)

Marker	Detection rate (%)		Relative methylation level		
	Control	NPC	Control	NPC	*P*
BILF2	34/145 (23.45)	174/202 (86.14)	−1.22 ± 3.89	9.56 ± 7.82	<0.001
ITGA4	1/145 (0.69)	130/202 (64.36)	−17.07 ± 1.32	−9.42 ± 5.86	<0.001
IMPA2	0/145 (0.00)	147/202 (72.77)	−17.12 ± 1.18	−10.55 ± 4.36	<0.001
ITPKB	4/145 (2.76)	147/202 (72.77)	−16.93 ± 1.77	−9.45 ± 5.16	<0.001
PI9	0/145 (0.00)	113/202 (55.94)	−17.12 ± 1.18	−9.55 ± 6.58	<0.001
AMIGO2	3/145 (2.07)	152/202 (75.25)	−17.00 ± 1.43	−8.49 ± 5.31	<0.001
VAV3	0/145 (0.00)	97/202 (48.02)	−17.12 ± 1.18	−10.56 ± 6.66	<0.001

Based on the significant differences in relative methylation levels between groups, diagnostic methods for NPC were constructed. Diagnosis was performed using a binary classification method. According to the above results, the EBV marker BILF2 was methylated in the majority of NPC samples, whereas they remained unmethylated in the most control samples. Thus, a relative methylation level of 0 was used as the cutoff value (COV): When the relative methylation level was >0, the predicted result was considered positive; otherwise, it was negative. For host methylation markers, methylated products were almost undetectable in control samples but were detectable in most NPC samples. Thus, for quality-qualified samples, if the methylated products of host genes were amplified, the results were considered positive, indicating a positive diagnosis. As shown in Table 3, the AUC of the diagnostic method for the EBV marker BILF2 was 0.890, with a sensitivity of 82.18% and a specificity of 95.86%. Among the diagnostic methods constructed by host markers, AMIGO2 (AUC = 0.866, sensitivity = 75.25%, specificity = 97.93%) and IMPA2 (AUC = 0.864, sensitivity = 72.77%, specificity = 100.00%) had relatively higher AUC. The VAV3 marker had the worst performance with an AUC of 0.740, with a sensitivity of 48.02%. Overall, the specificity of each diagnostic methods exhibited good specificity but relatively lower sensitivity.

**Table 3.** Performance of single-indicator diagnostic methods on the training set (*n* = 347)

Diagnostic method	AUC (95% CI)	Predict	Group		Sensitivity	Specificity
			NPC	Control	(%)	(%)
BILF2	0.890 (0.859, 0.921)	+	166	6	82.18	95.86
		−	36	139		
ITGA4	0.818 (0.785, 0.852)	+	130	1	64.36	99.31
		−	72	144		
IMPA2	0.864 (0.833, 0.895)	+	147	0	72.77	100.00
		−	55	145		
ITPKB	0.850 (0.817, 0.884)	+	147	4	72.77	97.24
		−	55	141		
PI9	0.780 (0.745, 0.814)	+	113	0	55.94	100.00
		−	89	145		
AMIGO2	0.866 (0.834, 0.898)	+	152	3	75.25	97.93
		−	50	142		
VAV3	0.740 (0.706, 0.775)	+	97	0	48.02	100.00
		−	105	145		

To explore the complementary effects between EBV and host methylation markers, we constructed NPC diagnostics by combining markers from both sources (Table 4). A positive diagnosis for any of the markers was considered positive, while negative diagnosis for both markers was considered negative. Compared to the BILF2 diagnostic method, the diagnostic method combining BILF2 with ITGA4, IMPA2, PI9, or VAV3 markers all showed improved detection performance. With specificity remaining unchanged, the sensitivity increased to 85.15%, 85.15%, 84.16%, and 84.16%, respectively. The IDI was 2.97%, 2.97%, 1.98%, and 1.98% (*P* < 0.05), respectively, indicating an improvement in predictive performance. Other diagnostic methods showed no significant difference compared to the single-marker diagnostic method BILF2. When combining BILF2 with 2 host markers, there was no further improvement in the diagnostic method’s performance (Table S2).

**Table 4.** Performance of diagnostic methods constructed by combining EBV marker BILF2 with a single host marker in the training set

Diagnostic method	Sensitivity	Specificity	AUC	IDI	*P*
	(%)	(%)	(95% CI)	(95% CI)	
BILF2	82.18	95.86	0.890	Ref	-
			(0.859, 0.921)		
BILF2 | ITGA4	85.15	95.86	0.905	0.0297	0.0131
			(0.876, 0.935)	(0.0062, 0.0532)	
BILF2 | IMPA2	85.15	95.86	0.905	0.0297	0.0131
			(0.876, 0.935)	(0.0062, 0.0532)	
BILF2 | ITPKB	85.15	94.48	0.898	0.0159	0.3023
			(0.867, 0.929)	(−0.0143, 0.0461)	
BILF2 | PI9	84.16	95.86	0.900	0.0198	0.0439
			(0.870, 0.930)	(5e−04, 0.0391)	
BILF2 | AMIGO2	84.16	95.17	0.897	0.0129	0.2824
			(0.866, 0.927)	(−0.0106, 0.0364)	
BILF2 | VAV3	84.16	95.86	0.900	0.0198	0.0439
			(0.870, 0.930)	(5e−04, 0.0391)	

### The performance of diagnostic methods in the validation cohort

**Fig. 5.**
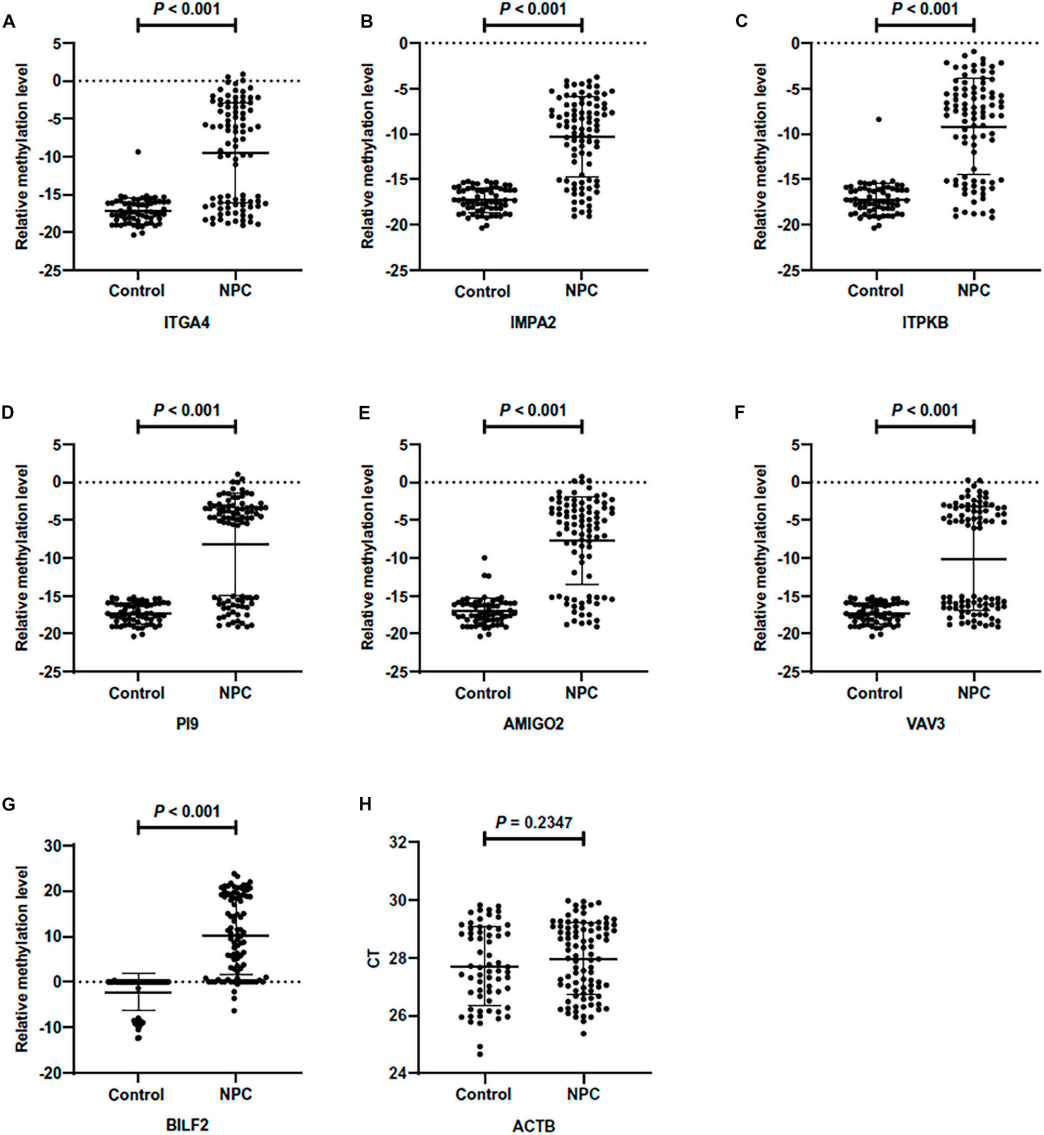
Comparison of relative methylation levels of methylation markers [ITGA4 (A), IMPA2 (B), ITPKB (C), PI9 (D), AMIGO2 (E), VAV3 (F), and BILF2 (G)] between case-controls in the validation set and comparison of CT values of internal reference gene ACTB (H).

In validation cohort, a total of 194 subjects were recruited including 74 controls and 120 NPC patients. Among them, 155 brushing samples (64 controls and 91 NPC) were quality-qualified and included in the analysis. There were significant differences in the relative methylation levels of each marker between groups, consistent with the results in the training cohort (Fig. 5 and Table 5). The performance of the diagnostic methods in the validation set showed that the BILF2 diagnostic method had an AUC of 0.893, with a sensitivity of 80.22% and a specificity of 98.44%. When combining BILF2 and IMPA2 markers, the AUC increased to 0.915, with an IDI of 4.40%, maintaining the specificity at 98.44% and increasing the sensitivity to 84.62%. However, the improvement in IDI for the diagnostic method combining BILF2 with ITGA4, PI9, or VAV3 markers was not statistically significant (*P* > 0.05) (Table 6).

**Table 5.** Candidate marker methylation detection in the validation set (*n* = 155)

Marker	Detection rate (%)		Relative methylation level		
	Control	NPC	Control	NPC	*P*
BILF2	17/64 (26.56)	77/91 (84.62)	−2.25 ± 4.10	10.18 ± 8.44	<0.001
ITGA4	1/64 (1.56)	55/91 (60.44)	−17.16 ± 1.68	−9.49 ± 6.60	<0.001
IMPA2	0/64 (0.00)	68/91 (74.73)	−17.28 ± 1.36	−10.31 ± 4.39	<0.001
ITPKB	1/64 (1.56)	68/91 (74.73)	−17.17 ± 1.74	−9.18 ± 5.29	<0.001
PI9	0/64 (0.00)	58/91 (63.74)	−17.28 ± 1.36	−8.20 ± 6.72	<0.001
AMIGO2	3/64 (4.69)	68/91 (74.73)	−17.05 ± 1.80	−7.75 ± 5.80	<0.001
VAV3	0/64 (0.00)	46/91 (50.55)	−17.28 ± 1.36	−10.06 ± 6.87	<0.001

**Table 6.** Performance of diagnostic methods constructed by combining EBV marker BILF2 with a single host marker in the validation set

Diagnostic method	Sensitivity	Specificity	AUC	IDI	*P*
	(%)	(%)	(95% CI)	(95% CI)	
BILF2	80.22	98.44	0.893	Ref	-
			(0.849, 0.937)		
BILF2 | ITGA4	85.71	96.88	0.913	0.0393	0.1700
			(0.871, 0.955)	(−0.0168, 0.0955)	
BILF2 | IMPA2	84.62	98.44	0.915	0.044	0.0419
			(0.875, 0.956)	(0.0016, 0.0863)	
BILF2 | ITPKB	83.52	96.88	0.902	0.0173	0.4784
			(0.858, 0.946)	(−0.0306, 0.0653)	
BILF2 | PI9	82.42	98.44	0.904	0.022	0.1550
			(0.862, 0.946)	(−0.0083, 0.0523)	
BILF2 | AMIGO2	82.42	93.75	0.881	−0.0249	0.4187
			(0.831, 0.930)	(−0.0852, 0.0355)	
BILF2 | VAV3	81.32	98.44	0.899	0.011	0.3173
			(0.856, 0.942)	(−0.0105, 0.0325)	

### The methylation levels of markers in early-stage NPC samples

**Fig. 6.**
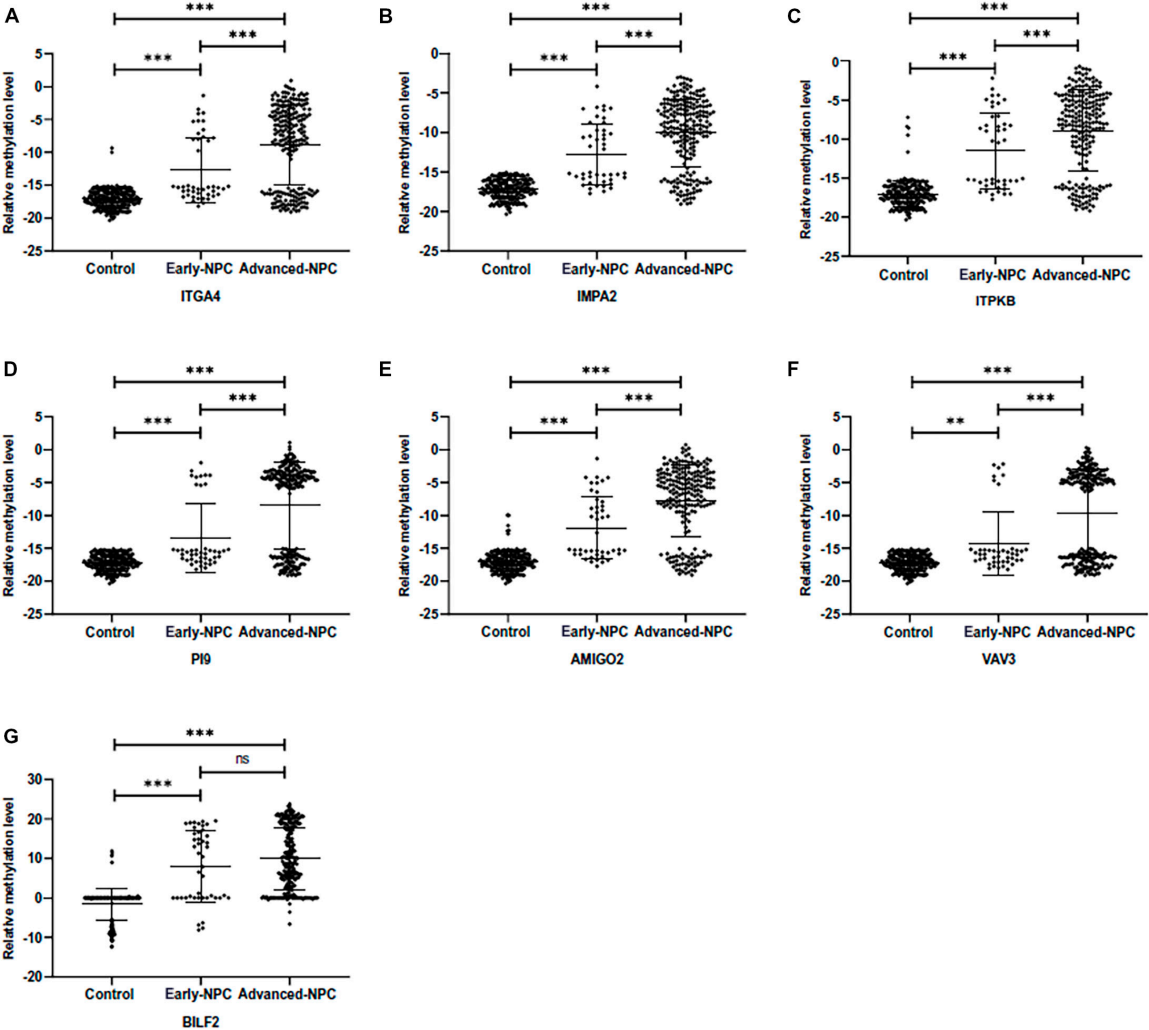
Relative methylation levels of methylation markers [ITGA4 (A), IMPA2 (B), ITPKB (C), PI9 (D), AMIGO2 (E), VAV3 (F), and BILF2 (G)] in early and advanced NPC samples.

Among all the blind brushing samples included in this study, 44 were from early-stage NPC patients, while the remaining samples were from advanced-stage NPC patients. As shown in Fig. 6, the relative methylation levels of all candidate markers were elevated in samples from early-stage patients. For host-derived markers, the relative methylation levels in samples from advanced-stage NPC patients were significantly higher than those from early-stage NPC patients, while there was no difference for the EBV methylation marker BILF2. In detection of early-stage NPC, the diagnostic method combining BILF2 with IMPA2 had a sensitivity of 68.18%.

## Discussion

Here, we identified NPC methylation biomarkers from both EBV and host source in blind nasopharyngeal brushing samples. Then, we trained the diagnostic method in the training cohort of 347 samples and performed validation in an independent cohort of 155 samples. When combining EBV-derived BILF2 and host source IMPA2 markers, the performance of diagnostic method increased, maintaining the specificity at 98.44% and increasing the sensitivity to 84.62%.

Compared to the nasopharyngeal brush sampling method, the blind brush sampling does not require the guidance of endoscope, making it more convenient for sample collection and better suited for implementation and promotion in community settings. In our previous study, we detected EBV DNA load and EBV Cp region methylation in both brushing samples and blind brushing samples. The results showed that there was no significant change in Cp methylation level between endoscopy-guided brushing samples and blind brushing samples, suggesting the feasibility of blind brushing samples [20]. In this study, we further explore the methylation biomarker in the blind brushing samples.

EBV is the first human tumor virus to be identified and is associated with the development and progression of various cancers, including NPC. EBV spreads in the population mainly in the form of saliva transmission. Changes in epigenetic modifications influence the expression patterns of EBV throughout its 3 life cycle stages. Previous study has revealed that EBV in the oral cavity of NPC patients exhibited hypermethylation, while in vast majority of control samples, EBV remained unmethylated [15]. In addition, methylation modifications of EBV, whether derived from plasma or nasopharyngeal brushing samples, also varied in patients with different tumors [14,21]. Here, we performed EBV capture and methylation sequencing on blind brushing samples from NPC and control subjects. An EBV methylation marker located on the BILF2 gene was identified, which exhibited better diagnostic performance compared to previous marker Cp (Table 1 and Fig. 1). Previously, we also found significant methylation differences in the BILF2 gene between NPC and nasal natural killer/T cell lymphoma [14]. It seems that the changes in BILF2 methylation modifications in NPC are unique and may be related to the mechanism by which the EBV contributes to the development of NPC. BILF2 is a gene expressed in the late lytic phase of EBV. It encodes a glycoprotein with an N-linked, gp78/55 [22]. However, the function of this gene is not yet clear and requires further research to explore.

Despite the diagnostic method including only the EBV methylation marker BILF2 achieving a specificity of 98.44%, its sensitivity (80.22%) still needed further improvement. Blind brush sampling reduced the amount of cellular material collected including EBV DNA due to the lack of endoscopic guidance, which in turn limited the sensitivity of the diagnostic method that included only EBV methylation markers [20]. Therefore, we explored the host-derived methylation markers. Many studies have explored the diagnostic value of abnormal methylation of TSGs in NPC, including RASSF1A, CDKN2A, and DAPK1. Most studies have employed the methylation-specific PCR (MSP) method, determining NPC by comparing the detection of methylated product bands. Despite many studies reporting abnormal hypermethylation of these genes in NPC, the results vary significantly among different studies. The detection rate of methylated CDKN2A products in NPC groups ranges from 22% to 67%, DAPK1 from 20% to 89%, and RASSF1A from 29% to 91%, indicating substantial heterogeneity among samples from different sources [10,11,23–34]. Thus, in the current study, we used methylation array data to rescreen methylation markers in NPC.

Based on the analysis of the GEO database, DMRs annotated on 8 genes were screened out (Figs. 2 and 3). Among them, the methylation difference of 6 DMRs was successfully validated in the training and validation cohorts, including ITGA4, IMPA2, ITPKB, PI9, AMIGO2, and VAV3 (Figs. 4 and 5). In the majority of NPC samples, these genes were detected as methylated, whereas in the control samples, they remained almost entirely unmethylated (Tables 2 and 5). ITGA4 has been previously reported to have higher methylation rates in NPC tissues compared to noncancerous tissues [35,36]. In addition, a study has indicated that ITPKB is a hub gene for NPC, showing hypermethylation and low expression in tumor tissues [37]. The remaining 4 genes, including IMPA2, PI9, AMIGO2, and VAV3, are newly identified differentially methylated genes in NPC. The roles of these genes in NPC occurrence are worth exploring in the future. When diagnosing NPC with a single methylation marker, host-derived markers were not as effective as the EBV-derived marker (Table 3). However, when combining markers BILF2 and IMPA2, the sensitivity of the diagnostic method improved, suggesting that host-derived markers had a complementary effect on EBV-derived markers (Tables 4 and 6).

The methylated products of host-derived markers showed almost no amplification in control samples and amplification in most NPC samples. Therefore, when host markers were included, the specificity of the diagnostic method was not affected, while the sensitivity was improved to some extent. For early-stage NPC patients, the sensitivity of the diagnostic method combining BILF2 with IMPA2 was 68.18%, which was lower than that for advanced-stage patients. However, when using tissue or endoscopy- guided brushing samples for detection (Table S3), the sensitivity could be increased to over 90% (Table S4). This suggested that the performance of this diagnostic method could be further improved by increasing sampling accuracy. In addition, approximately 22% of samples were excluded due to inadequate sampling quality. In the future, the following methods may be used to handle these samples. On the one hand, for subjects with negative EBV detection in blind brushing samples, repeat sampling and testing could be conducted using a secondary sampling method. On the other hand, using alternative detection methods could also improve the testing performance on samples, such as more sensitive digital PCR, or enzyme-based detection methods that do not rely on bisulfite modification.

This study also has some limitations. First, this study only conducted testing on blind brushing samples and did not compare them with serological antibody or plasma EBV DNA markers. In the future, paired sampling could be conducted to compare the diagnostic performance of each indicator and explore their combined effects. Second, this study only examined the application value of differentially methylated genes in NPC detection. Differences at the epigenetic modification may indicate differences at the gene expression level or reflect changes in the function of related regulatory factors. Further biological experiments could be conducted to explore the molecular mechanisms of each differentially methylated gene in the pathogenesis of NPC or EBV-associated diseases.

### Conclusion

In summary, this study explored NPC methylation markers from both host and EBV perspectives, and constructed a brush-based diagnostic method by combining markers from both sources. Compared to previous methods that only included EBV markers, this diagnostic method further improved the diagnostic performance.

## Materials and Methods

### Study design and participants

**Fig. 7.**
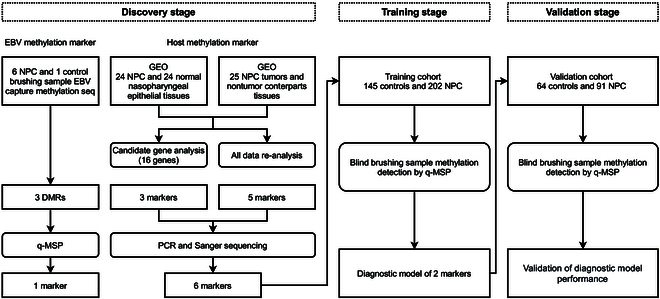
Workflow indicating study design.

The workflow of this study is illustrated in Fig. 7. In the discovery stage, EBV-derived candidate methylation markers were obtained from EBV capture and methylation sequencing of blind brushing samples. The DMR located on the BILF2 gene was selected. By analyzing GEO data in conjunction with blind brushing sample PCR amplification and PCR product Sanger sequencing, 6 host source methylation markers were identified. In the training stage, a total of 452 subjects were recruited, including 173 controls and 279 NPC patients. Among them, 347 samples were valid (CT_ACTB_ < 30) and subjected to detection. The methylation differences of candidate markers between groups were validated in the training samples, and diagnostic methods were constructed. In the validation stage, 194 subjects were recruited, including 74 controls and 120 NPC patients. A total of 155 samples from 64 controls and 91 NPC patients were valid. The performance of NPC diagnostic method was further validated in the validation set. All participants were recruited at Sun Yat-sen University Cancer Center. The inclusion criteria for the NPC group were as follows: (a) pathological diagnosis of NPC and (b) newly diagnosed cases, with no NPC-related treatment at the time of sample collection. The inclusion criteria for the non-NPC control group were as follows: (a) no history of NPC and (b) diagnosed as not having NPC through nasal pharyngeal tissue biopsy or imaging examination. The exclusion criteria for participants were as follows: (a) presence of mental or cognitive disorders, such as dementia, deafness, and comprehension difficulties; (b) complete loss of behavioral ability and difficulties in movement; (c) presence of other head and neck tumors; and (d) pregnant or breastfeeding women. The demographic characteristics of the subjects included in this study were demonstrated in Table S5.

### Nasopharyngeal blind brush sampling and processing

Nasopharyngeal blind brush sampling was used in this study, which means sampling without guidance of a nasal endoscope as previously described [20]. In brief, prior to biopsy sampling, trained personnel inserted a nasopharyngeal brush (Copan Diagnostics, Murrieta, CA) deeply into the subjects’ nasopharynx through both nostrils and rotated it several times against the nasopharyngeal epithelium to collect the sample. Then, the brush tip was cut and immersed in 1 ml of RNAlater (Invitrogen, Carlsbad, CA) and finally stored at −80 °C until DNA extraction. Genomic DNA was extracted from the nasopharyngeal blind brushing samples using Chemagic Star workstation (Hamilton, Perkin Elmer, Waltham, USA). Extracted DNA was further bisulfate modified using the EZ DNA Methylation-Gold kit (Zymo Research, CA, USA).

### EBV genome capture and methylation sequencing

The DNA libraries were constructed from 8 NPC and 7 control nasopharyngeal brushing samples and subjected to EBV-targeted bisulfite sequencing. Briefly, the DNA pools were first subjected to random fragmentation, end repair, A-tailing, and methyl-adaptor ligation. Hybridization capture (VariantBaits Target Enrichment Library Prep Kit, LCBio Tech, Hangzhou, China) was then performed using the EBV-targeting single-stranded DNA probes (Integrated DNA Technologies, Coralville, IA, USA) to enrich the EBV genomes. The captured DNA was then subjected to bisulfite conversion (EZ DNA Methylation-Gold Kit, Zymo Research, Irvine, CA, USA) and PCR amplification (SureSelect Methyl-Seq PCR Kit, Agilent Technologies, Santa Clara, CA, USA). The preprocessed libraries were sequenced using the NovaSeq 6000 platform (Illumina, San Diego, CA, USA). Six NPC samples and one control sample were successfully sequenced.

### Quantitative PCR analysis

The EBV DNA load was measured by quantitative PCR method. The targeted BamHI-W region amplification primers and dual-labeled hybridization probes were used to assess the EBV DNA load in the samples, consistent with a previously established method [38]. Plasmid DNA containing the target region in serial dilution (10^3^, 10^4^, 10^5^, 10^6^, and 10^7^ copies/μl) was used to establish the standard curve for absolute quantification. Each PCR was set up in a volume of 8 μl, containing 4 μl of PCR master mix, 1 μl of primers, 0.2 μl of probe, 0.8 μl of ddH_2_O, and 2 μl of DNA template. PCR was performed in LightCycler 480 under the following conditions: a denaturation step for 5 min at 95 °C, then 45 cycles of 95 °C for 15 s, 60 °C for 30 s, and 72 °C for 15 s, and then a final cooling step for 5 min at 72 °C. Samples without amplification were considered negative.

The relative methylation level of methylation marker was measured by quantitative methylation-specific PCR (qMSP). The bisulfite-converted DNA was used as the template for amplification. The reaction of qMSP was performed in LightCycler 480 under the following conditions: a denaturation step for 5 min at 95 °C, then 50 cycles of 95 °C for 15 s and 58 °C for 60 s, and then a final cooling step for 30 s at 40 °C. For the detection of EBV DNA methylation markers, a dual-probe detection method targeting both methylated and unmethylated products was used, with each reaction volume of 10 μl containing 5 μl of PCR master mix, 0.8 μl of primers, 0.4 μl of methylated probe, 0.4 μl of unmethylated probe, 2.4 μl of ddH_2_O, and 1 μl of bisulfate-modified DNA. The CT (cycle threshold) value for methylated probe (CTm) and CT value for unmethylated probe (CTu) can be obtained. If CTm or CTu cannot be obtained, a value of CT = 45 was used to fill. If both values cannot be obtained, the sample was considered undetectable. The relative methylation level of the EBV DNA methylation marker was reflected using ΔCT and compared between groups, where ΔCT = −(CTm − CTu). For undetectable samples, use ΔCT = 0.

For the detection of host genomic DNA methylation markers, only a single probe targeting methylation products was used. Each reaction contained 5 μl of PCR master mix, 0.8 μl of primers, 0.4 μl of methylated probe, 2.8 μl of ddH_2_O, and 1 μl of bisulfate-modified DNA. The reference gene β-actin (ACTB) was used to assess brushing samples’ quality, with samples having an ACTB amplification CT < 30 considered valid. The relative methylation level of the host genomic methylation marker was calculated using −(CTm − CT_ACTB_). The primers and probes used in this study were shown in Table S6.

### Identification of methylation markers

The EBV DNA methylation markers were obtained by comparing EBV DNA methylation sequencing results between the NPC and control groups. Two GEO datasets (GSE52068 and GSE62336) were used to screen the host genomic DNA methylation markers. The GSE52068 dataset provided the Illumina 450K methylation array data from 24 NPC tissues and 24 control tissues [39], while GSE62336 contained Illumina 450K methylation array data from 25 NPC tissues and their adjacent normal tissues [8]. Two strategies, including candidate strategy and reanalysis strategy, were used for the initial screening of host methylation markers. PCR amplification (8 NPC and 8 control blind brushing samples) and Sanger sequencing of the products were used for further candidate marker selection. Candidate markers were validated in an expanded sample set using the qMSP method.

### Statistical analysis

The statistical analyses were performed using R software, version 4.1.2. For descriptive analysis, continuous variable with a normal distribution was tested for statistical differences using the *t* test, while continuous variables with a skewed distribution were analyzed using the Mann–Whitney test. Categorical variables were analyzed using the χ^2^ test. *P* < 0.05 was considered statistically significant.

Receiver operating characteristic (ROC) analysis was conducted, and the performance of the diagnostic model was evaluated by AUC, sensitivity, and specificity at the optimal COV or at a predetermined COV. IDI was used to compare the predictive performance of 2 diagnostic methods.

## Data Availability

The data that support the findings of this study are available from the corresponding authors upon reasonable request.
