# Supplementary material for: Blind Brush Biopsy: Quantification of Epstein–Barr Virus and Its Host DNA Methylation in the Detection of Nasopharyngeal Carcinoma
Source: Research (Wash D C). 2024 Sep 24;7:0475. doi: 10.34133/research.0475 (PMC11420652; doi:10.34133/research.0475)
Supplement: Supplementary 1 — Figs. S1 to S5 Tables S1 to S6 [file research.0475.f1.docx]

Full title: Blind Brush Biopsy: Quantification of EBV and Its Host DNA Methylation in the Detection of Nasopharyngeal Carcinoma

Short title: Bind Brush Biopsy in the Detection of NPC

Caoli Tang^1,2,^†, Xizhao Li^1,^†, Yumeng Zhang^1,2^, Ting Zhou^1^, Xiaojing Yang^1^, Ying Liao^1^, Tongmin Wang^1^, Yongqiao He^1^, Wenqiong Xue^1^, Weihua Jia^1,2,*^ and Xiaohui Zheng^1*^

^1^ State Key Laboratory of Oncology in South China, Guangdong Key Laboratory of Nasopharyngeal Carcinoma Diagnosis and Therapy, Guangdong Provincial Clinical Research Center for Cancer, Sun Yat-sen University Cancer Center, Guangzhou 510060, P. R. China.

^2^ School of Public Health, Sun Yat-sen University, Guangzhou 510080, China.

†These authors contributed equally to this work.

^*^Address correspondence to: Xiaohui Zheng; [zhengxh@sysucc.org.cn](mailto:zhengxh@sysucc.org.cn) and Weihua Jia; [jiawh@sysucc.org.cn](mailto:jiawh@sysucc.org.cn)

**Supplementary Figures**

**Fig. S1.** Scatter plot of methylation levels of CpG sites within the regions 10,243-10,608 bp, 137,999-138,149 and 11,029-11,197 bp in NPC and control nasopharyngeal brushing samples from EBV-targeted bisulfite sequencing results.


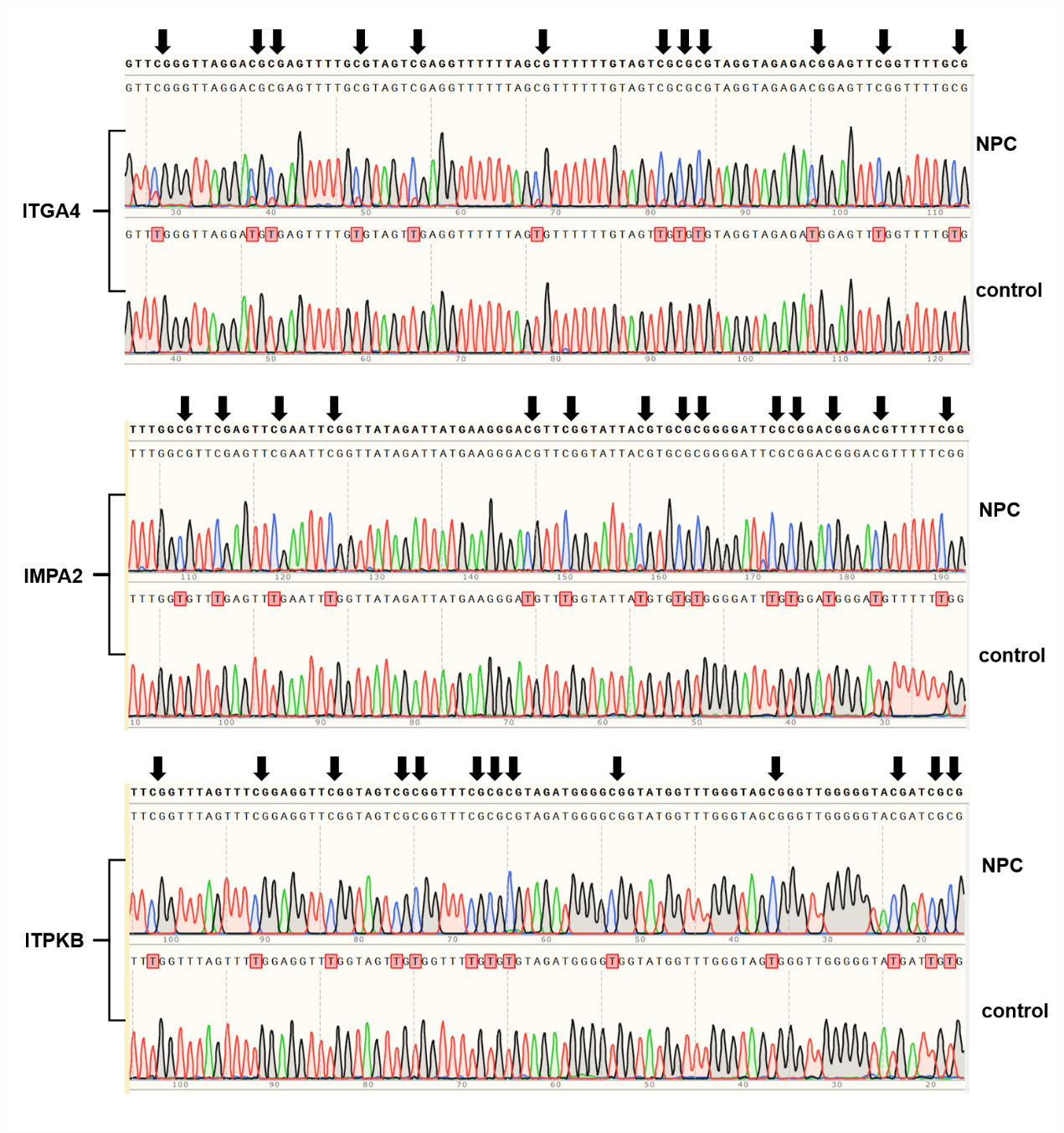


**Fig. S2.** The Sanger sequencing results of the amplified products from ITGA4, IMPA2, and ITPKB genes regions. The figure illustrated partial results of the amplified products from one case sample and one control sample. Similar results were obtained for the remaining samples and regions. The red peaks in the figure represent thymine (T) according to Sanger sequencing, while the blue peaks represent cytosine (C). The black arrows pointed to the location of the CpG site within the displayed region. The detection results indicated that at the CpG site within the displayed region, the NPC sample showed detection as CpG, while the control sample showed detection as TpG.


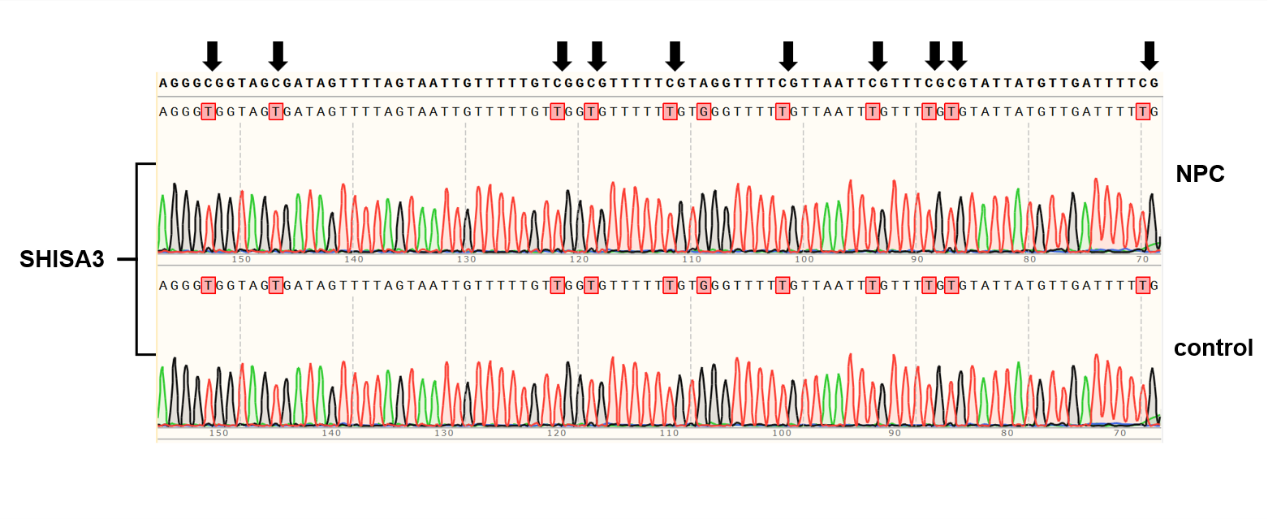


**Fig. S3.** The Sanger sequencing result of the amplified products from SHISA3 genes regions. The detection results indicated that at the CpG site within the displayed region, both the NPC and control samples amplified products were detected as TpG.


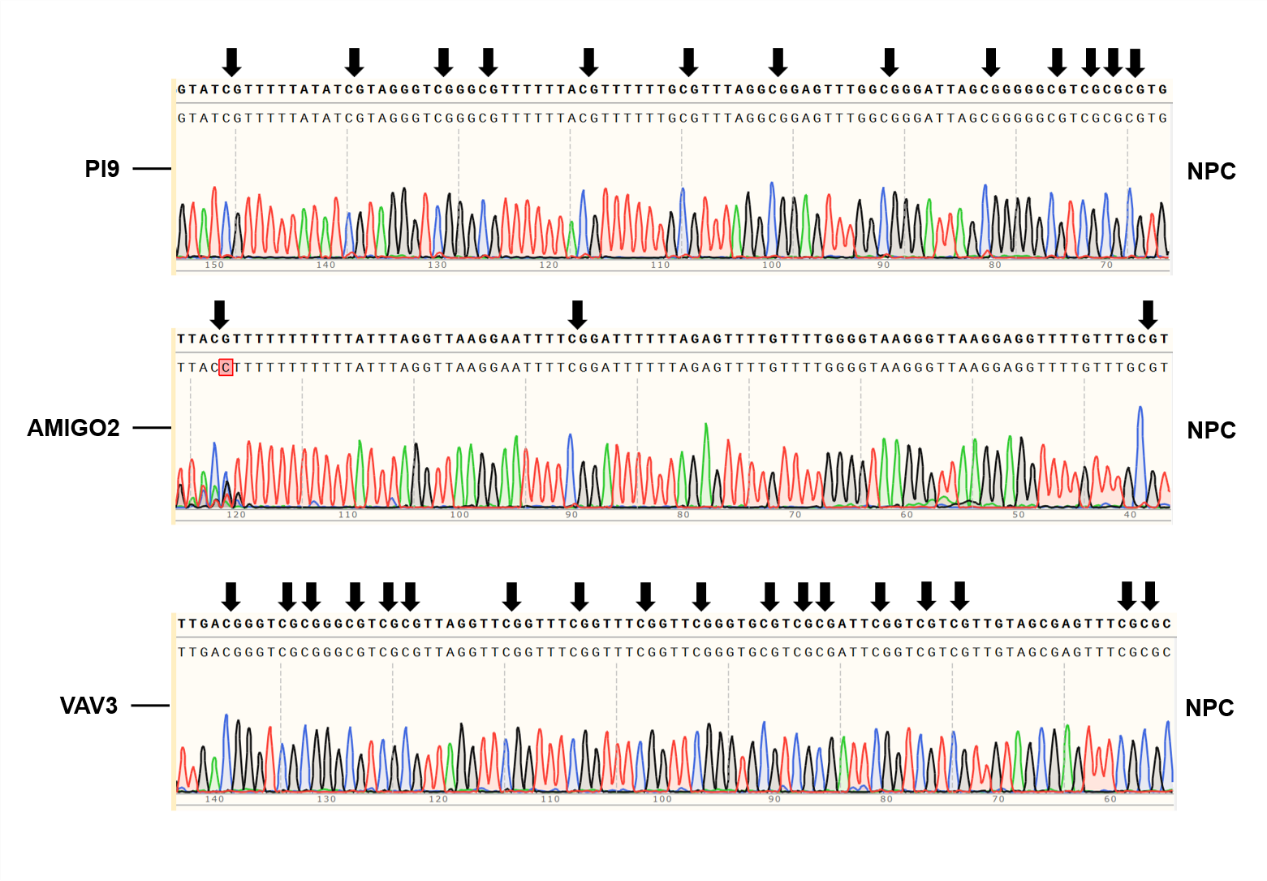


**Fig. S4.** The Sanger sequencing result of the amplified products from PI9, AMIGO2, and VAV3 genes regions. The detection results indicated that at the CpG site within the displayed region, the NPC sample amplified products were detected as CpG. The sequencing of methylated products in the control samples failed.


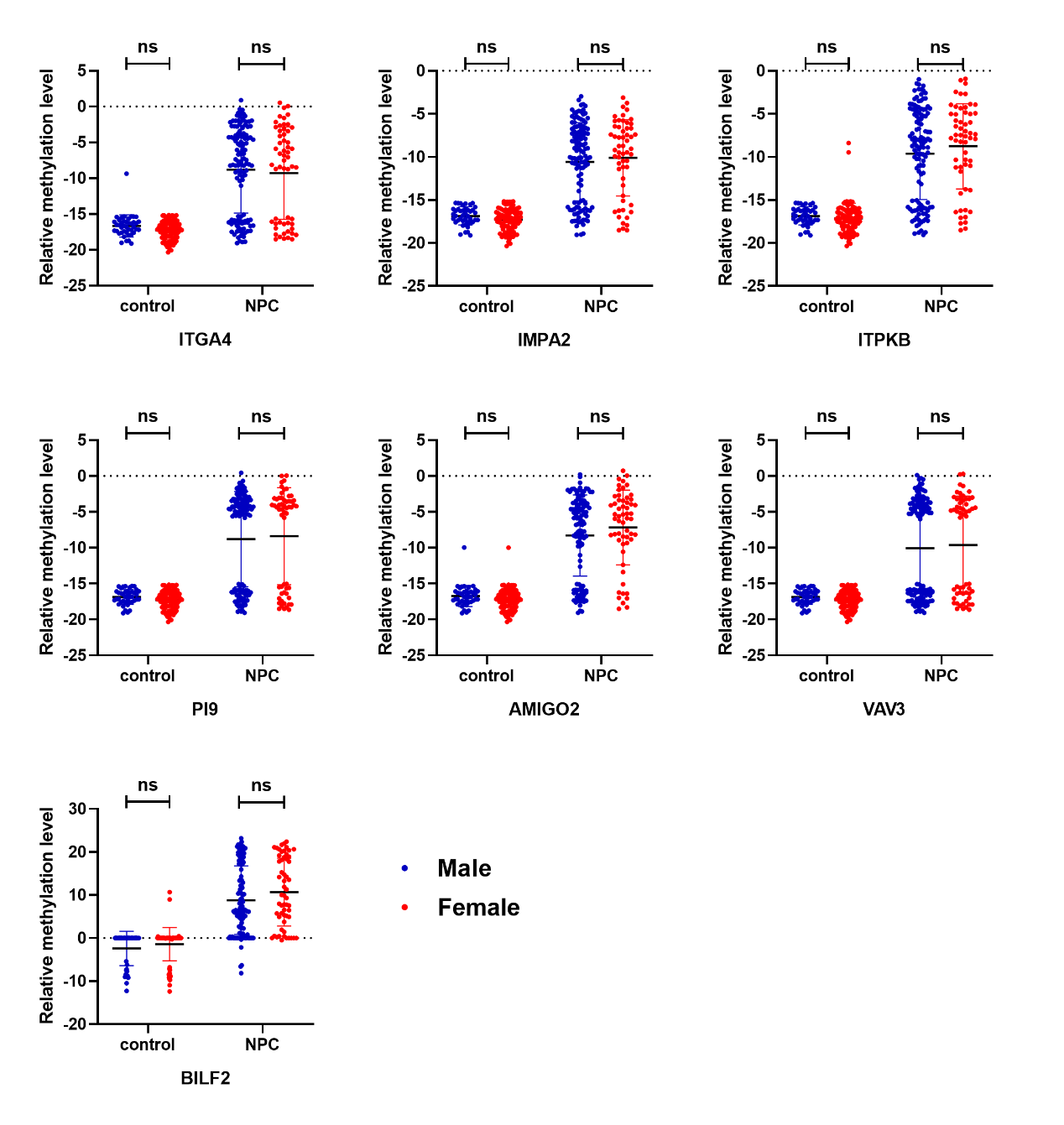


**Fig. S5.** Comparison of relative methylation levels of candidate genes between case and control groups, stratified by gender, within the training set.

**Supplementary Tables**

| **Table S1.** Detection of methylated products of candidate genes in case and control groups within the training set, stratified by gender. | | | | | | |
| --- | --- | --- | --- | --- | --- | --- |
| Marker | control (%) | | | NPC (%) | | |
|  | Male | Female | *P* | Male | Female | *P* |
| BILF2 | 15/51(29.41) | 19/94(20.21) | 0.9999 | 127/144(88.19) | 47/58(81.03) | 0.5531 |
| ITGA4 | 0/51(0.00) | 1/94(1.06) | 0.9999 | 95/144(65.97) | 35/58(60.34) | 0.9999 |
| IMPA2 | 0/51(0.00) | 0/94(0.00) | 0.9999 | 105/144(72.92) | 42/58(72.41) | 0.9999 |
| ITPKB | 1/51(1.96) | 3/94(3.19) | 0.9999 | 105/144(72.92) | 42/58(72.41) | 0.7671 |
| PI9 | 0/51(0.00) | 0/94(0.00) | 0.4976 | 82/144(56.94) | 31/58(53.45) | 0.7576 |
| AMIGO2 | 0/51(0.00) | 3/94(3.19) | 0.9999 | 107/144(74.31) | 45/58(77.59) | 0.9129 |
| VAV3 | 0/51(0.00) | 0/94(0.00) | 0.2969 | 70/144(48.61) | 27/58(46.55) | 0.2681 |

| **Table S2.** Performance of diagnostic methods constructed by combining EBV marker BILF2 with two host markers in the training set. | | | | | | | | | | | |
| --- | --- | --- | --- | --- | --- | --- | --- | --- | --- | --- | --- |
| Diagnostic method | Sensitivity (%) | Specificity (%) | AUC | IDI^a^ | *P*^a^ | IDI^b^ | *P*^b^ | IDI^c^ | *P*^c^ | IDI^d^ | *P*^d^ |
| BILF2 \| ITGA4 | 85.15 | 95.86 | 0.905 | ref | ref | - | - | - | - | - | - |
| BILF2 \| IMPA2 | 85.15 | 95.86 | 0.905 | - | - | ref | ref | - | - | - | - |
| BILF2 \| PI9 | 84.16 | 95.86 | 0.900 | - | - | - | - | ref | ref | - | - |
| BILF2 \| VAV3 | 84.16 | 95.86 | 0.900 | - | - | - | - | - | - | ref | ref |
| BILF2 \| ITGA4 \| IMPA2 | 86.14 | 95.86 | 0.910 | 0.0099 | 0.1563 | 0.0099 | 0.1563 | 0.0198 | 0.0439 | 0.0198 | 0.0439 |
| BILF2 \| ITGA4 \| ITPKB | 85.64 | 94.48 | 0.901 | -0.0088 | 0.4175 | -0.0088 | 0.4954 | 0.0011 | 0.9348 | 0.0011 | 0.9348 |
| BILF2 \| ITGA4 \| PI9 | 85.64 | 95.86 | 0.908 | 0.0050 | 0.3173 | 0.0050 | 0.5644 | 0.0149 | 0.0817 | 0.0149 | 0.0817 |
| BILF2 \| ITGA4 \| AMIGO2 | 85.64 | 95.17 | 0.904 | -0.0019 | 0.8187 | -0.0019 | 0.8598 | 0.0080 | 0.4684 | 0.0080 | 0.4684 |
| BILF2 \| ITGA4 \| VAV3 | 85.64 | 95.86 | 0.908 | 0.0050 | 0.3173 | 0.0050 | 0.5644 | 0.0149 | 0.0817 | 0.0149 | 0.0817 |
| BILF2 \| IMPA2 \| ITPKB | 85.64 | 94.48 | 0.901 | -0.0088 | 0.4954 | -0.0088 | 0.4175 | 0.0011 | 0.9348 | 0.0011 | 0.9348 |
| BILF2 \| IMPA2 \| PI9 | 85.15 | 95.86 | 0.905 | 0.0000 | 1.0000 | 0.0000 | NaN | 0.0099 | 0.1563 | 0.0099 | 0.1563 |
| BILF2 \| IMPA2 \| AMIGO2 | 85.15 | 95.17 | 0.902 | -0.0069 | 0.5683 | -0.0069 | 0.3173 | 0.0030 | 0.7595 | 0.0030 | 0.7595 |
| BILF2 \| IMPA2 \| VAV3 | 85.15 | 95.86 | 0.905 | 0.0000 | 1.0000 | 0.0000 | NaN | 0.0099 | 0.1563 | 0.0099 | 0.1563 |
| BILF2 \| ITPKB \| PI9 | 85.15 | 94.48 | 0.898 | -0.0138 | 0.2499 | -0.0138 | 0.2499 | -0.0039 | 0.7450 | -0.0039 | 0.7450 |
| BILF2 \| ITPKB \| AMIGO2 | 85.15 | 93.79 | 0.895 | -0.0207 | 0.1333 | -0.0207 | 0.1333 | -0.0108 | 0.4332 | -0.0108 | 0.4332 |
| BILF2 \| ITPKB \| VAV3 | 85.15 | 94.48 | 0.898 | -0.0138 | 0.2499 | -0.0138 | 0.2499 | -0.0039 | 0.7450 | -0.0039 | 0.7450 |
| BILF2 \| PI9 \| AMIGO2 | 84.16 | 95.17 | 0.897 | -0.0168 | 0.1639 | -0.0168 | 0.0870 | -0.0069 | 0.3173 | -0.0069 | 0.3173 |
| BILF2 \| PI9 \| VAV3 | 84.16 | 95.86 | 0.900 | -0.0099 | 0.3173 | -0.0099 | 0.1563 | 0.0000 | NaN | 0.0000 | NaN |
| BILF2 \| AMIGO2 \| VAV3 | 84.16 | 95.17 | 0.897 | -0.0168 | 0.1639 | -0.0168 | 0.0870 | -0.0069 | 0.3173 | -0.0069 | 0.3173 |
| ^a^ Compare to method BILF2+ITGA4; ^b^ Compare to method BILF2+IMPA2; ^c^ Compare to method BILF2+PI9; ^d^ Compare to method BILF2+VAV3. | | | | | | | | | | | |

| **Table S3.** Amplification of methylation markers in tissue and endoscopy-guided NP brushing samples from early-stage NPC. | | | | | | | | |
| --- | --- | --- | --- | --- | --- | --- | --- | --- |
| Sample type | ID | Amplification CT value | | | | | | BILF2^a^ |
|  |  | ITGA4 | IMPA2 | ITPKB | PI9 | AMIGO2 | VAV3 |  |
| Tissue | T1 | - | - | - | - | - | - | 15.85 |
|  | T2 | 31.85 | 34.55 | 33.85 | 31.22 | 31.82 | 31.30 | 20.79 |
|  | T3 | - | - | - | 32.14 | - | - | 17.45 |
|  | T4 | 32.65 | 34.68 | 32.71 | 31.67 | 32.44 | 32.78 | 20.05 |
|  | T5 | - | 36.47 | - | 32.27 | 32.71 | - | 21.21 |
|  | T6 | - | 34.55 | 31.56 | 30.98 | 31.24 | 31.52 | 20.17 |
|  | T7 | 32.79 | 34.22 | 31.13 | 31.99 | - | 31.48 | 22.52 |
|  | T8 | - | - | - | - | - | - | 0.00 |
|  | T9 | - | 35.08 | 32.40 | 31.95 | 32.97 | - | 18.14 |
|  | T10 | 28.33 | 30.16 | 35.03 | 28.01 | 27.94 | 28.31 | 23.45 |
|  | T11 | 31.92 | 34.55 | 31.36 | 30.27 | 32.01 | 29.95 | 23.12 |
| Endoscopy-guided brushing | E1 | 33.47 | 36.33 | 34.00 | 32.19 | 34.33 | 32.86 | 18.80 |
|  | E2 | 33.17 | 34.85 | 32.83 | 32.09 | 32.98 | - | 19.09 |
|  | E3 | 35.60 | 39.32 | 37.69 | - | 37.73 | - | 13.73 |
|  | E4 | - | - | - | - | - | - | 0.53 |
|  | E5 | 34.28 | 37.45 | 35.93 | - | 35.29 | 32.93 | 15.67 |
|  | E6 | - | - | - | - | - | - | 0.40 |
|  | E7 | - | - | - | - | 38.92 | - | 13.21 |
|  | E8 | - | 42.35 | 41.12 | - | 40.95 | - | 14.21 |
|  | E9 | - | - | 39.40 | - | 39.00 | - | 11.76 |
|  | E10 | 37.18 | 39.71 | 38.71 | 35.97 | 35.55 | 35.43 | 18.30 |
| ^a^ The relative methylation level of BILF2; - No amplification. | | | | | | | | |

| **Table S4.** Comparison of sensitivity for various detection methods across different sample types from early-stage NPC. | | | |
| --- | --- | --- | --- |
| Detection method | Sensitivity | | |
|  | Blind brushing (n = 44) | Endoscopy-guided brushing (n = 10) | Tissue (n = 11) |
| BILF2 | 29(65.91%) | 10(100.00%) | 10(90.91%) |
| ITGA4 | 15(34.09%) | 5(50.00%) | 5(45.45%) |
| IMPA2 | 21(47.73%) | 6(60.00%) | 8(72.73%) |
| ITPKB | 22(50.00%) | 7(70.00%) | 7(63.64%) |
| PI9 | 10(22.73%) | 3(30.00%) | 9(81.82%) |
| AMIGO2 | 21(47.73%) | 8(80.00%) | 7(63.64%) |
| VAV3 | 7(15.91%) | 3(30.00%) | 6(54.55%) |
| BILF2 \| IMPA2 | 30(68.18%) | 10(100.00%) | 10(90.91%) |

| **Table S5.** Demographic characteristics of training cohort and validation cohort subjects. | | | | | | | |
| --- | --- | --- | --- | --- | --- | --- | --- |
|  | Training cohort | | |  | Validation cohort | | |
|  | control | NPC | *P* |  | control | NPC | *P* |
| N | 145 | 202 |  |  | 64 | 91 |  |
| Age (years,‾X±S) | 46.54±12.58 | 47.16±12.01 | 0.6446 |  | 48.83±13.58 | 48.83±11.54 | 0.2674 |
| Gender (%) |  |  | <0.001 |  |  |  | <0.001 |
| Male | 51 | 144 |  |  | 19 | 65 |  |
| Female | 94 | 58 |  |  | 45 | 26 |  |
| Tumor stage |  |  |  |  |  |  |  |
| I | - | 9 |  |  | - | 0 |  |
| II | - | 25 |  |  | - | 10 |  |
| III | - | 70 |  |  | - | 35 |  |
| IV | - | 83 |  |  | - | 31 |  |
| NA^a^ | - | 15 |  |  | - | 15 |  |
| ^a^ Data not available | | | | | | | |

| **Table S6.** Primers and probes sequence used in this study. | |
| --- | --- |
| Name | Sequence (5' to 3') |
| BamHI-W-F | CCCAACACTCCACCACACC |
| BamHI-W-R | TCTTAGGAGCTGTCCGAGGG |
| BamHI-W-probe | FAM-CACACACTACACACACCCACCCGTCTC-TAMRA |
| Cp-F | GAGTGTTATTTTTGGAATAGTAG |
| Cp-R | TTAAACTCTCTTATTAACTATAATC |
| Cp-M-probe | FAM-TGAATTTTGTTGGCGGGAGAAGGA-BHQ1 |
| Cp-U-probe | HEX-TGAATTTTGTTGGTGGGAGAAGGA-BHQ1 |
| BILF2-F | TGGAAGTAGTTACGGTTAAGG |
| BILF2-R | TCACCGCCCATACAAATACTA |
| BILF2-M-probe | FAM-ATTTTCGGGAGTGTATTTTCGGTTT-BHQ1 |
| BILF2-U-probe | HEX-TATTTTTGGGAGTGTATTTTTGGTTTA-BHQ1 |
| Intergenic region-F | TTGTTTTAGGTTTATTTTAGGAG |
| Intergenic region-R | AATAAAACAAACAACTAATATACTT |
| Intergenic region-M-probe | FAM-TCGGGTTAGTATTTTATTAGTTTAACG-BHQ1 |
| Intergenic region-U-probe | HEX-TTGGGTTAGTATTTTATTAGTTTAATGG-BHQ1 |
| ITGA4-F | GCGAGTTTTGCGTAGTCGAG |
| ITGA4-R | TCTCCGAATACGAACCGCTAA |
| ITGA4-probe | FAM-TAGGTAGAGACGGAGTTCGGTTTTGC-BHQ1 |
| IMPA2-F | ACGACGTTTAGCGTTTAGCGG |
| IMPA2-R | CCGCGCACGTAATACCGAAC |
| IMPA2-probe | FAM-TGTTGGTTTAGTCGTTTTTGGCGTTCG-BHQ1 |
| ITPKB-F | TTCGGCGTTTTCGGTTTAGTTTC |
| ITPKB-R | AACTAAACCCGCGATCGTACC |
| ITPKB-probe | FAM-AGGTTCGGTAGTCGCGGTTTCG-BHQ1 |
| PI9-F | TCGTCGCGGATTTTCGTTAGG |
| PI9-R | CTCTAACCTCGACCACCACGCG |
| PI9-probe | FAM-TTTATATCGTAGGGTCGGGCGTTT-BHQ1 |
| AMIGO2-F | CGTTTTTTCGTTTAGTTTTAAGTC |
| AMIGO2-R | ACGTAACCTATACGACCGCCGC |
| AMIGO2-probe | FAM-GGGATACGTTTAGTTATCGAGGGAG-BHQ1 |
| VAV3-F | AGTAGGAGTCGCGGTTGACGG |
| VAV3-R | AATCGAAAACCTATACGCGACAAC |
| VAV3-probe | FAM-TTCGGTTTCGGTTTCGGTTCGGGTG-BHQ1 |
| ACTB-F | TGGTGATGGAGGAGGTTTAGTAAGT |
| ACTB-R | AACCAATAAAACCTACTCCTCCCTTAA |
| ACTB-probe | FAM-ACCACCACCCAACACACAATAACAAACACA-BHQ1 |
